# Health sciences and medical librarians conducting research and their experiences asking for co-authorship

**DOI:** 10.5195/jmla.2022.1485

**Published:** 2022-10-01

**Authors:** Jamie E. Bloss, Kerry Sewell, Jana Schellinger, Amanda Haberstroh

**Affiliations:** 1 blossj19@ecu.edu, Laupus Health Sciences Library, East Carolina University, Greenville, NC.; 2 browderk@ecu.edu, Laupus Health Sciences Library, East Carolina University, Greenville, NC.; 3 jlschellinger@ehc.edu, E&H Library, Emory & Henry College School of Health Sciences, Marion, VA.; 4 haberstroha17@ecu.edu, Laupus Health Sciences Library, East Carolina University, Greenville, NC.

**Keywords:** Authorship, librarians, medical librarians, health science librarians, research ethics, intradisciplinary scholarship, negotiation, professional support

## Abstract

**Objective:**

Health sciences librarians frequently engage in scholarly publication, both with other librarians undertaking intradisciplinary scholarship, and increasingly as members of research teams centered in other disciplines. We sought to assess the emotional and institutional context of authorship among health sciences librarians, including emotions experienced during authorship negotiation, the frequency with which authorship is denied, and the correlation of perceived support from supervisors and the research community with the number of publications produced.

**Methods:**

342 medical and health sciences librarians took an online survey of 47 questions regarding emotions experienced when asking for authorship, denial of authorship, if they have been given authorship without asking, and the extent to which they felt supported to conduct research in their current job.

**Results:**

Authorship negotiation creates varied and complex emotions among librarians. The emotions reported differed when negotiating authorship with librarian colleagues and when negotiating authorship with professionals in another field. Negative emotions were reported when asking either type of colleague for authorship. Respondents reported feeling mostly supported and encouraged by their supervisors, research communities, and workplaces. Nearly one quarter (24.4%) of respondents reported being denied authorship by colleagues outside of their departments. Perceived research appreciation and support by the research community is correlated with the total number of articles or publications produced by librarians.

**Conclusion:**

Authorship negotiation among health sciences librarians involves complex and frequently negative emotions. Denial of authorship is frequently reported. Institutional and professional support appear to be critical to publication among health sciences librarians.

## INTRODUCTION

Published research is increasingly collaborative, involving intradisciplinary teams or including researchers from other disciplines, with the number of authors per paper indicating the increasingly collaborative nature of research [1, 2, 3, 4]. The reasons for the rise in multi-authored papers is likely manifold, involving issues related to specialization, technology, and publish-or-perish pressures [5]. Its heightened place in employee evaluations, tenure and promotion processes, as well as grant funding decisions, are ample indications of the extent to which the pursuit of authorship is vital to the careers of many scientists. While authorship expectations vary by discipline in terms of the norms for format (monograph vs. journal article), volume of publication, and authorship order, publication itself is a shared goal across the disciplines.

Among librarians, while authorship may be similarly required for career advancement at some institutions, it is more frequently described as conferring other benefits such as advancing knowledge within the profession, establishing the value of the library to stakeholders, promoting critical and scientific thinking, and leading to changes to library practices [6, 7]. Authorship on collaborative teams is also described as something that has intrinsic benefits, with librarians as coauthors reporting feeling more fulfilled, perceiving a better reputation among researchers in non-Library and Information Science (LIS) departments, and greater job satisfaction [8].

This emphasis on the benefits of research engagement beyond career advancement may reflect the role and status of librarians within institutions. Librarians may not have faculty status in many universities, access to tenure in institutions where faculty status is given [9], or work as solo librarians where time constraints due to multi-faceted duties mean that both time and collaboration networks are limited [10]. Having roles not requiring or allowing for research engagement may shift motivations from those related to job stability and advancement to more service-oriented motivations for the individual library and the profession. Additionally, even beyond the uniquely challenging situation for solo librarians, time to dedicate to research is a frequently reported barrier for librarians [11, 7]. For librarians who do engage in publication, institutional and professional community support may be critically important [11], with research showing “positive correlations between knowledge creation and productivity, engagement and perceived support from the supervisors and researcher community” [12].

The differing motivations and contexts for engaging in publishing notwithstanding, evidence indicates that librarians are more frequently engaged in supporting and participating in collaborative research [13]. Although collaboration has increased, the collaboration may largely be intraprofessional. The amount of interprofessional collaboration is reported to be increasing but is still low [14, 15], with one study of the prevalence of co-authorship for librarians in scholarly higher education (HE) and multidisciplinary teaching and learning (TL) journals over a 12-year period, finding that only 1.38% were authored or co-authored by a librarian [16].

The apparent low level of interprofessional collaboration is surprising in the health sciences, given the strong recommendation for a librarian within systematic review (SR) and meta-analysis teams, which typically originate from non-library disciplines. Despite being involved in systematic reviews from an early date and being shown to improve the quality of a systematic review [17], a relatively small number of published systematic reviews include librarian coauthors or acknowledge librarian involvement [17, 18, 19]. Even in cases when a librarian is involved in a systematic review in accordance with national and international standards for systematic reviews, denial of authorship occurs. One study indicates that while librarians are often or always granted an author role on a systematic review or meta-analysis, 15.6% of librarians (or roughly three out of twenty) participating in systematic reviews report being often or sometimes denied authorship [20]. The denial of authorship is notably unrelated to any lack of awareness of prominent authorship criteria among health sciences librarians on SR teams. Limited research indicates that librarians are aware of authorship criteria [8], namely those of the International Committee of Medical Journal Editors (ICMJE) [21] and that those criteria, as well as the Institute of Medicine's 2011 Standards for Systematic Reviews [22], are explicitly referenced on health sciences libraries web pages on systematic review support services [23].

Health sciences and medical librarians in the US often take part in SR research, but there has been little research on authorship credit for collaborative work done outside of the realm of SR research. Similarly, there has been little research on the affective experience of health sciences and medical librarians who have been denied or offered authorship credit in relation to the composition of the research team. The challenges that health sciences librarians face when collaborating on systematic reviews indicate barriers that may arise for librarians working on collaborative research teams more broadly; namely, institutional, peer, and community support for research, as well as the rates of denial of authorship. Librarians may also face challenges in securing credit for their research contributions due to lack of formal or informal training in the negotiation process inherent in collaborative authorship. These three issues may all lead to differing affective experiences in authorship negotiations when working intraprofessionally and interprofessionally.

Anecdotally, the affective nature of authorship negotiation experiences among librarians is evidenced by frequent discussion at discipline-specific training sessions regarding how best to ask for authorship and therefore a seat at the scholarly table, while navigating the associated anxiety and distress involved in the authorship negotiation process. Articles have been published about librarians and impostor syndrome, librarians and anxiety related to technology or “technostress,” as well as librarians and teaching anxiety, but there is a paucity of published literature on the affective experience of negotiating authorship credit on publications [24, 25, 26, 27, 28, 29, 30]. This same paucity is noted outside of LIS literature. This paper will further explore the affective experience of librarians as researchers and co-authors, with an examination of that experience based upon the disciplinary composition of research teams, and if perceived support and encouragement from the LIS research community or from supervisors is correlated to the number of papers librarians have written.

## RESEARCH AIMS

This research aims to address the following:

1. Do medical or health sciences librarians feel supported and encouraged by their supervisors and peers to conduct research and to what degree?
2. What emotions do medical or health sciences librarians experience when asking for authorship credit and to what degree?
3. Have medical or health sciences librarians been denied authorship outright and how often?
4. For medical or health sciences librarians, are factors such as appreciation or support from supervisors and peers correlated to the total number of articles published?
5. Do demographic factors or other factors such as years of experience in the field or faculty status affect medical or health sciences librarians asking for authorship?

These questions allow the exploration of librarians' experiences as co-authors with other librarians and with those outside of their departments. The findings of this study will elucidate authorship experiences among health sciences librarians and may have implications for the profession related to the pre-service and in-service training offered to health sciences librarians, such as training on authorship norms and ethics, and negotiation. A better understanding of the affective nature of authorship negotiations, the organizational contexts that promote it, and the training needs that emerge from results of this study would better equip librarians and librarian supervisors to address authorship needs.

## METHODS

A survey was iteratively developed by four health sciences librarians (Appendix A). A statistician and research data librarian were consulted about appropriate analytical models according to question types. For the purpose of the study, authorship consisted of the following: “1) substantial contribution to the work and 2) accountability for the work that was done and its presentation in a publication” [31]. We used the questions from a survey on perceptions of the research community using a Likert scale which was originally validated from Pyhalto's work with doctoral students, then adapted for post-docs, which also used a 1–7-point scale [12]. We also used the Discrete Emotions Questionnaire to assess the emotional experience when asking for authorship [32]. The other sections of the survey, which included demographic questions and information about the librarian's career thus far, were determined by the research team. We also included a section of the survey on self-described communication style using a list of the four basic styles of communication [33]. The survey was pre-tested by ten researchers in various fields including library and information studies, public health, psychology, and human sexuality studies to gain feedback on the flow and workability of the survey, that the research questions would be addressed by the survey, and for assistance with wording on gender and other demographic questions. Feedback was incorporated into the survey before it was sent to the East Carolina University (ECU) Institutional Review Board (IRB) for approval; the survey was granted exempt status (UMCIRB 21-001144).

The Qualtrics link for the survey was distributed to 25 listservs pertaining to medical and health sciences librarians inclusive of the Medical Library Association (MLA) listserv and 13 MLA caucus listservs, 12 regional MLA chapter listservs, and the Association of Academic Health Sciences Libraries (AAHSL) listserv. Language was also developed for word-of-mouth distribution and both that script and the email language were approved by the ECU IRB. We decided not to include academic librarians working in university or college settings who did not work with health sciences patrons in this study because of the differences in experiences that might occur between a generalist librarian's experience to that of a liaison to health sciences or medical departments. The survey was open to responses from July 6, 2021, to August 6, 2021, and one reminder email was sent after the first two weeks of the survey being open.

Data was coded in Excel and analyzed using SPSS statistical software version 28. Data visualizations were created in Excel and Google Spreadsheets. Data is available via the Open Science Framework (OSF) data repository at https://osf.io/dgkxu/. Statistics were collected and analyzed as planned and included descriptive statistics for all data points. Other statistical analyses including crosstabs with chi-squared, T-test, bivariate correlation with confidence intervals, and bivariate linear regression were used as indicated in the results section. Data was averaged for emotions questions and the mean was used to aid in comparison. Table 1 describes the tests run for each of our research questions.

**Table 1:** Statistical analysis used for each research question

Research Question Number	Research Question	Statistical Analysis Used	Explanation of Statistical Tests
Question 1	How supported and encouraged do medical or health sciences librarians feel by their supervisors and peers to conduct research?	Descriptive statistics	Descriptive statistics-Summarizes data as a representation of the whole
Question 2	Do factors such as appreciation or support from supervisors and peers affect librarians as they seek authorship opportunities and are the total number of articles or publications related to that support?	Descriptive statistics	Bivariate correlation-shows relationships between two variables.
Question 3	What emotions do medical or health sciences librarians experience asking for authorship and to what degree?	Bivariate correlations with Confidence Interval	Bivariate linear regression – shows relationships between two variables assuming one influences the other
Question 4	Do demographic factors or other factors such as years of experience in the field or faculty status affect medical, health sciences librarians asking for authorship?	Bivariate linear regressions	Independent 2-tailed t-tests – examines whether two population means are equal
Question 5	Have medical or health sciences librarians been denied authorship outright and how often?	Descriptive statistics	

## RESULTS

The survey received 342 responses. Table 2 shows the breakdown of selected demographic information of the participants. For full descriptive information of survey participants, please refer to the Open Science Framework (OSF) data repository (https://osf.io/dgkxu/).

**Table 2:** Selected Participant Demographics

Gender	Female	224	66.1%
	Male	41	12.1%
	Genderqueer	4	1.2%
	Did Not Report	70	20.6%
Sex	Female	220	64.9%
	Male	41	12.1%
	Did Not Report	78	23.0%
Race or Ethnicity	White	230	67.8%
	Asian	10	2.9%
	Black	5	1.5%
	Puerto Rican	1	0.3%
	2 or More Races	7	2.1%
	Did Not Report	86	25.4%
Hispanic	Hispanic	20	5.9%
	Not Hispanic	245	70.2%
	Did Not Report	73	21.5%

### Research and Supervisor Support

Respondents reported feeling mostly supported and encouraged by their supervisors, research communities, and workplaces (Table 3).

**Table 3:** Support Librarians Experience

	Strongly Agree	Agree	Somewhat Agree	Neither Agree nor Disagree	Somewhat disagree	Disagree	Strongly disagree
I receive encouragement and personal attention from my supervisor(s).	41.1% (n=123)	26.8% (n=80)	15.4% (n=46)	4.7% (n=14)	4.3% (n=13)	4.7% (n=14)	3.0% (n=9)
I work in a library or setting that encourages research production.	32.1% (n=96)	26.4% (n=79)	19.1% (n=57)	6.0% (n=18)	6.0% (n=18)	6.4% (n=19)	4.0% (n=12)
I feel that my supervisor(s) appreciate(s) my work.	44.6% (n=133)	28.5% (n=85)	12.4% (n=37)	4.7% (n=14)	3.7% (n=11)	3.4% (n=10)	2.7% (n=8)
I can openly discuss any problems related to research with my supervisor(s).	30.6% (n=91)	27.3% (n=81)	16.5% (n=49)	8.4% (n=25)	5.7% (n=17)	6.1% (n=18)	5.4% (n=16)
I feel accepted by my research community.	17.1% (n=51)	35.8% (n=107)	23.4% (n=70)	14.0% (n=42)	4.7% (n=14)	4.0% (n=12)	1.0% (n=3)
I feel appreciated by my supervisor(s).	44.6% (n=133)	27.9% (n=83)	12.8% (n=38)	3.0% (n=9)	4.0% (n=12)	5.4% (n=16)	2.3% (n=7)
I feel that the other members of my research community appreciate my work.	21.5% (n=64)	37.9% (n=113)	21.1% (n=63)	15.8% (n=47)	2.3% (n=7)	1.3% (n=4)	0.0% (n=0)
There is a good sense of collegiality among the researchers I interact with.	26.1% (n=78)	45.5% (n=136)	18.1% (n=54)	6.7% (n=20)	2.3% (n=7)	1.3% (n=4)	0.0% (n=0)
I receive encouragement and support from the other researchers.	19.5% (n=58)	32.7% (n=111)	24.8% (n=74)	13.4% (n=40)	2.7% (n=8)	2.0% (n=6)	0.3% (n=1)

### Emotions medical or health sciences librarians experience asking for authorship

**Figure 1:**
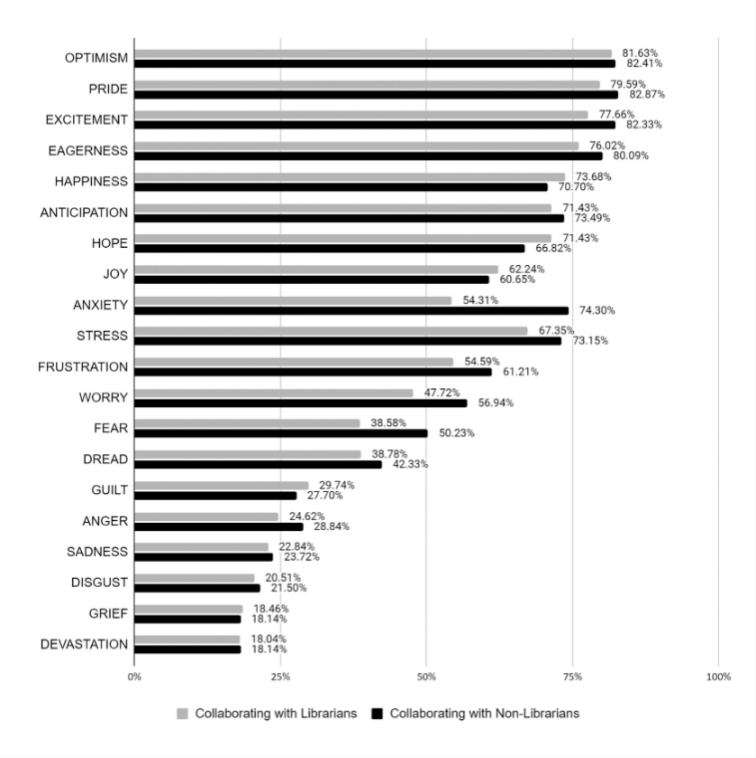
Librarian Emotions When Negotiating Authorship

Librarians experience a variety of emotions when asking for authorship, both with other library colleagues and with researchers outside of their departments (Figure 1, data available in Appendix B). A series of independent t-tests were performed to compare each emotion in librarians asking for authorship when publishing with other library colleagues, and librarians publishing with researchers outside of their departments. Tests showed that librarians who published with other library colleagues had significantly higher emotional scores when asking for authorship in the following areas: fear, sadness, dread, grief, happiness, and joy. Librarians who published with researchers outside their department had significantly higher emotional scores when asking for authorship in the areas of anxiety, frustration, and pride (Table 4). Significance was determined based on p<.05.

**Table 4:** Independent Samples Test for Equality of Means

	t	df	Sig. (2-tailed)	Mean Difference	Std. Error Difference	95% Confidence Interval of the Difference	
						Lower	Upper
FEAR	7.597	392.687	.000	33.629	4.426	24.927	42.332
ANXIETY	-6.607	388.148	.000	-29.291	4.433	-38.008	-20.575
SADNESS	7.465	409.408	.000	32.506	4.354	23.946	41.066
WORRY	-1.111	410	.267	-5.260	4.736	-14.570	4.050
DREAD	6.559	395.874	.000	29.387	4.480	20.579	38.196
FRUSTRATION	-5.378	396.408	.000	-24.414	4.540	-33.339	-15.489
ANGER	-.750	407	.454	-3.037	4.050	-10.998	4.924
DISGUST	-.592	408	.554	-2.246	3.797	-9.711	5.219
DEVASTATION	.034	407	.973	.126	3.725	-7.196	7.448
GRIEF	9.496	404.861	.000	39.563	4.166	31.373	47.753
STRESS	1.276	409	.203	5.473	4.291	-2.962	13.908
GUILT	-.454	406	.650	-1.972	4.349	-10.521	6.576
HAPPINESS	2.318	476.560	.021	8.239	3.554	1.256	15.223
EXCITEMENT	1.333	411	.183	4.920	3.692	-2.338	12.178
PRIDE	-3.873	402.435	.000	-16.265	4.200	-24.521	-8.009
JOY	4.046	378.846	.000	16.913	4.181	8.693	25.133
EAGERNESS	1.651	410	.100	6.227	3.772	-1.188	13.643
OPTIMISM	-1.886	410	.060	-7.393	3.920	-15.099	.313
ANTICIPATION	-1.068	408	.286	-4.641	4.347	-13.186	3.905
HOPE	-9.585	404.780	.000	-40.999	4.278	-49.408	-32.590

This chart provides the data for the appropriate variance based on a p-value of 0.05 (Equal variance > 0.05 and not equal variance < 0.05). For a full chart with both variances for each data point, please refer to the Open Science Framework (OSF) data repository (https://osf.io/dgkxu/).

## DENIAL OF AUTHORSHIP

For authors who published or worked on publications with other librarians or library staff, 61.5% of respondents had been offered authorship on the publication without having to ask. 38.5% said they had not been offered authorship. Only 4.9% said they had been refused authorship by other library colleagues, and for those who were refused authorship, most had only been refused one time. One respondent was refused 4 times. For authors who published or worked on publications with other researchers who were not librarians or library staff, 75.6% were offered authorship without having to ask. 24.4% had not been offered authorship. 24.4% said they had been refused authorship by colleagues who were not librarians. Most had been refused 1 or 2 times over the course of their careers, while some respondents reported being refused authorship on publications between 3-20 times.

### Research productivity and perceived support and appreciation from the supervisors/workplace and the research community

We ran bivariate correlations with confidence intervals (Table 5) to determine correlation between the number of publications a respondent had as first author or any other spot in the author order and the statement questions about encouragement and support from supervisors, workplaces, and the research community.

**Table 5:** Bivariate Correlations with CI and Bivariate Linear Regressions Tests

Statement questions about encouragement and support from supervisors, workplaces, and the research community	Number of Publications as First Author (correlations)	Number of Publications not First Author (correlations)	Number of Publications as First Author (Significance = p≤.05)	Number of Publications not First Author (Significance = p≤.05)
I receive encouragement and personal attention from my supervisor(s).	-0.044	0.055	0.555	0.392
I work in a library or setting that encourages research production.	0.113	0.154	0.129	0.017
I feel that my supervisor(s) appreciate(s) my work.	-0.104	0.035	0.162	0.587
I can openly discuss any problems related to research with my supervisor(s).	-0.055	0.059	0.46	0.363
I feel accepted by my research community.	0.096	0.245	0.197	<.001
I feel appreciated by my supervisor(s).	-0.109	0.024	0.144	0.708
I feel that the other members of my research community appreciate my work	0.194	0.193	0.009	0.003
There is a good sense of collegiality among the researchers I interact with.	0.059	0.148	0.432	0.021
I receive encouragement and support from the other researchers.	0.162	0.145	0.029	0.024

None of the correlations were particularly strong and could be described as weak to very weak. However, looking at those who were lead author versus lower-ranked co-authors on a collaborative paper, for those who were lead authors, the statements: “I feel that the other members of my research community appreciate my work” and “I receive encouragement and support from the other researchers” were more strongly correlated with having authored more publications. For those who had publications as lower-ranked co-authors, the statements: “I feel accepted by my research community” and “I feel that the other members of my research community appreciate my work,” were more strongly correlated with having authored more publications in a team.

We ran exploratory statistics with a bivariate linear regression (Table 5) to determine if any of the support or encouragement variables were significant. Statistical significance was set as p<.05. The statements “I feel that the other members of my research community appreciate my work” and “I receive encouragement and support from the other researchers” were statistically significant for the respondents who had publications as first author. For those who had publications as author in any other author order spot, the following statements had a statistically significant impact on their number of publications: “I work in a library or setting that encourages research production,” “I feel accepted by my research community,” “I feel that the other members of my research community appreciate my work,” “There is a good sense of collegiality among the researchers I interact with,” and “I receive encouragement and support from the other researchers.”

### Factors that affect asking for authorship

We ran crosstabs analysis with chi-squared and found that race, ethnicity, gender, and sex were not significantly correlated to when participants had asked for authorship either with librarian colleagues or with others outside of their departments.

Total years of experience working in a library and years worked in a librarian position were not significant in either support from supervisors, research communities, or research encouragement, nor was it significant in asking for authorship, or being offered or refused authorship, except in the case of asking for authorship with non-librarian collaborators where total years of experience had significance.

The type of librarian or job designation held (i.e., liaison librarian, solo librarian, hospital librarian) or having faculty status, tenure, or being administrative staff did not have significance on whether other librarian collaborators offered authorship without them having to ask, asking for authorship with non-librarian collaborators, and refusal of authorship with non-librarian collaborators. However, someone's job role or if they were faculty were statistically significant in agreeing with the following statements: “I receive encouragement and personal attention from my supervisor(s),” “I work in a library or setting that encourages research production”, and “I can openly discuss any problems related to research with my supervisor(s).”

## DISCUSSION

Findings indicate that authorship experiences among health sciences librarians are influenced by the disciplinary composition of collaborative research teams, as well as institutional support for research engagement.

### Disciplinary composition of coauthor teams and librarian authorship

The disciplinary composition (librarian-only vs. collaborators from outside the profession, henceforth referred to as intraprofessional and interprofessional collaborations) of coauthor teams affected medical librarians' experiences of authorship in two ways: the emotions experienced when negotiating authorship and rates of denial of authorship.

Findings indicate that librarians express a wider array of negative emotions (sadness, dread, grief, fear) when asking for co-authorship on intraprofessional collaborations than when asking for co-authorship on interprofessional collaborations (anxiety, frustration). Although feelings of happiness and joy were significant for intraprofessional authorship negotiation experiences, it was notable that pride was more frequently reported when negotiating for authorship on interprofessional collaborations.

This study cannot elucidate the reasons for these differences in negative and positive emotions based upon the disciplinary composition of coauthor teams, but the findings may indicate that in-group (i.e., intraprofessional) vs. out of group (interprofessional) negotiations for authorship involve different dynamics and carry different implications for library professionals. Inclusion as a coauthor on intraprofessional teams may, for instance, validate one's belongingness within the profession and, more locally, the specific library if all members work in the same institution. This phenomenon corresponds with conceptions of relational capital as reflected in authorship, in which coauthoring papers reflects “trust, commitment, and reciprocity within the collective,” [34] in this case, within the collective of the LIS profession and within specific library systems. By contrast, inclusion as an author on interprofessional teams may be less about belonging and more about validation of the unique expertise and work that a librarian offers in interprofessional research endeavors, serving as a source of pride.

This latter posited phenomenon (coauthorship as a form of validation of unique expertise in interprofessional research teams) has not been explicitly studied and elaborated in literature on librarian authorship (or indeed, authorship more widely), though related issues of power and bias in interprofessional teams have been widely discussed [35]. This phenomenon of coauthorship as validation of unique expertise would be in line with understandings of professionals as those whose “identity and status … is not given or determined but is rather a precarious, contested formation constantly negotiated through discursive activity” [36] and in which professionals must work to convince others that their roles and contributions are unique and legitimate [37]. Library literature is indicative of the extent to which the recognition of professional legitimacy of librarians may be fraught [37, 38, 39], particularly in research endeavors [40, 41, 42]. In the context of authorship, limited research indicates the extent to which under-recognition of librarian contributions to research teams persists in higher education, with nearly 40% of librarian respondents to a survey on collaboration between librarians and other researchers participating together on systematic reviews reporting that researchers view them as PDF suppliers or administrative support personnel on research teams [20] rather than as research collaborators. Advice on publishing includes comments such as “be prepared to do the initial leg work, because your faculty liaisons may consider you a valued colleague, but not necessarily someone they would consider coauthoring an article with” [43]. The published literature suggesting that librarians' roles in research endeavors are underrecognized is, notably, tempered by other literature that suggests that, as librarians are increasingly embedded in credit-bearing courses and actively engaged in interprofessional research, faculty from other disciplines are coming to more highly value the role of librarians [44, 45, 39].

The disciplinary composition of potential coauthor teams also affects the rates of failed authorship negotiations (i.e., denial of author role), with significantly higher rates of failed negotiations for authorship on interprofessional manuscripts than intraprofessional manuscripts (24.4% vs. 4.9%, respectively). These findings may indicate one of two things: that librarians negotiating authorship roles on interdisciplinary teams are not perceived to adequately fulfill criteria for authorship that research collaborators are following (such as the rigorous International Committee of Medical Journal Editors (ICMJE) criteria) or that librarian expertise and credibility in research endeavors is sometimes underrecognized among researchers outside of the library profession, leading to higher rates of denial of authorship [21]. In examining authorship criteria, Borrego & Pinfields' 2020 study on authorship among librarians indicates that librarians are highly aware of ICMJE criteria, as well as other codified authorship criteria outside of the health sciences, but that librarians may sometimes have difficulty convincing other researchers that they fulfill the applicable authorship criteria [8].

With regards to the latter potential explanation, if true, librarians' perceptions of being “invisible, overlooked, and underestimated” [46] may affect authorship negotiations in two ways: if librarians' contributions are indeed undervalued in research endeavors, non-librarian researchers may be less likely to grant librarians an authorship role on a manuscript; or, librarians' internalized perceptions of their perceived value within the research enterprise may make them less able to self-advocate effectively for authorship roles. Limited research on the effects of power differences in authorship negotiations provides support for this [47, 48], along with opinion pieces on authorship for graduate students [49]. Outside of authorship contexts, research on how perceived status as well as gender- and race-based power differences affect negotiations find that those in low-status positions (either hierarchically or based upon gender and race/ethnicity) may have a harder time negotiating successfully and that perceived professional status, gender, and race interact in complex ways to affect both the behavior of negotiators and the outcomes of negotiation [50, 51]. The mechanism identified as influencing both behaviors and success in negotiations is the adherence to or violation of norms for gender, race, and hierarchical status and the ways that those in positions of power react to behaviors adhering to or violating those norms [52]. Further study is warranted on how librarians' demographics and their perceptions of their professional status affect their negotiation for authorship.

### Institutional support for research

Respondents reported high levels of support for research within their library or institution, with the mode of responses being ‘strongly agree’ to questions related to working in a library or setting that encourages research production, as well as supervisory encouragement and personal attention, appreciation of work, and openness to discussing research issues as they arise. The majority of respondents also indicated that they felt accepted, appreciated, encouraged, and supported by other researchers within a broader research community. In examining the mode of the responses to questions about librarians' perceptions of acceptance, appreciation, encouragement, and support within a broader research community, it is notable that the mode was ‘agree,’ and the second-highest response was ‘somewhat agree.’ The authors speculate that acceptance, appreciation, encouragement, and support within a broader research community may be more difficult for librarians to assess than the direct, codified feedback mechanisms that a supervisor and an institution provide about research engagement and production.

In examining the ways that faculty status for librarians may affect research engagement and production among health sciences librarians, we found faculty status was significant when examined in relation to the following statements: “I receive encouragement and personal attention from my supervisor(s)”, “I work in a library or setting that encourages research production”, and “I can openly discuss any problems related to research with my supervisor(s).” The data suggest institutional recognition of librarians as faculty members not only confers an expectation of engagement in research based on the status, but has an effect on library workplace culture, improving supervisory support for and communication about research. The extent to which faculty status affects scholarly engagement among librarians has been examined in a study by Laws, who found that librarians who have faculty status are more active in scholarly activity and may have an easier time asking for authorship if this status is given [53].

These findings are of note in considering the removal of faculty status among librarians at many institutions. In a recent survey of medical librarians, just 60.9% of respondents said that librarians at their institution hold faculty status [53]. Another study from 2016 showed that only 52% of U.S. research universities grant any kind of faculty status to librarians [54]. The authors acknowledge that librarians working outside academia may not have any option for faculty status and that some librarians may not desire to have faculty status or want to publish research on teams.

Support for research in and of itself within librarianship appears to be important even beyond faculty status. In the present study, higher perceived support for research within the librarian research community was correlated to more first authorship positions for librarians. The authors note that while faculty status may automatically confer an expectation of research, the support from both supervisors and colleagues across the profession is critical in promoting research engagement and authorship. Research emphasis and positive group climate are two of the factors associated with successful creation of institutional research environments [55], with collaborative inter-institutional networks further increasing scholarship. While the authors hold the role of professional and institutional research support to be critical for librarian authorship endeavors, the authors also acknowledge that librarians with more first author positions may report higher perceived support for research within the librarian community due to greater recognition of their work based on the authorship order.

## IMPLICATIONS

Implications for the library field include focusing on strategies and future educational opportunities to help librarians overcome negative emotions in asking for authorship for their contributions. Training opportunities related to authorship criteria and ethics as well as effective strategies for negotiation in research teams would be helpful. The authors also suggest continued efforts to provide research methods training for medical librarians to address a lacuna in methodological expertise that may leave librarians with lower levels of research literacy and concomitant feelings of insecurity in collaborative research teams. In a 2014 study [16], many librarians reported that they felt that they needed more research methodology training or had not received enough in their MLIS programs. This could also be a contributing factor to a fear of asking for authorship. This type of training and continuing education has been shown to influence how often librarians ask for authorship on systematic reviews, boosting the numbers of how many librarians asked for authorship after the training [56].

Related to library culture for research, several efforts could assist medical librarians in authorship experiences. Library associations could provide more programs focused on enhancing the research culture of libraries, geared toward both administrators and librarians, such as the MLA Research Training Institute [57]. Medical libraries could formalize mentorship models to support research engagement, systematic review participation, and authorship among early career health sciences librarians. Supervisors are noted as having a strong role to play in establishing mentorship models and encouraging collaborative research culture within their departments, but departmental colleagues could also contribute to fostering research and authorship among new colleagues through conscientious and purposeful efforts to include new librarians in LIS-focused research projects.

The findings related to the correlation between perceived support and appreciation by broader LIS research communities and first author positions for librarians indicate that more can be done to provide this type of support. Creating more opportunities for professional relationship-building opportunities and extra-institutional mentorship/mentee opportunities would potentially increase medical librarian authorship. Professional organizations could also improve LIS research culture by creating and running journal clubs focused on discussing LIS research. Lastly, while many caucuses in MLA celebrate and promote librarian research, more could be done to recognize librarians as researchers such as promoting members' publications via professional association websites or social media. Intraprofessional validation of librarians as researchers might help librarians feel more confident in their authorship negotiations within interprofessional research team contexts.

For individual librarians, additional ways to combat challenges to collaboration with other researchers include negotiating authorship upfront in the research process and becoming more involved with the research process [8]. Emphasizing clear and frequent communication, clarification of individuals' roles in the process, and methods for successfully conducting in-depth consultations to overcome the challenges of communication or misunderstandings of the librarian's role in a research project should be employed [20]. The authors note that some of the negative emotions librarians experience when asking for authorship could be influenced by past negative experiences, interactions, and microaggressions experienced with fellow librarians and other researchers, and therefore it cannot be placed fully on the librarian's shoulders to overcome these issues on their own when they are overlooked for authorship after significantly contributing to a work [58].

Further research, in the form of follow-up interviews with consenting participants, is planned, and will further inform practical recommendations for the field.

## LIMITATIONS

The demographic characteristics of respondents to this survey may be considered representative of the professional membership of the Medical Library Association (MLA) based upon a recent survey of MLA membership [59]. Sex/gender tracked closely with the MLA survey, though response rates for Asian/Asian American and Black medical librarians were slightly lower among our respondents (2.9% and 1.5% respectively, versus 6% and 6% in the MLA report). However, the insufficient representation of minority groups in our sample did not allow for an assessment of the effect of race, ethnicity, gender, or sex on asking for authorship. Given that authorship negotiations are often discussed as negotiations hinging upon “power differences, cross-cultural and cross-disciplinary assumptions” [60], demographic characteristics that form the basis of power differences and cross-cultural differences, particularly those arising from sexual and gender minority status or racial and ethnic minority status may affect authorship negotiations in ways that cannot be sufficiently elucidated by this study [51]. Additionally, based on MLA membership statistics as of 2019, the majority (95%) of the participants in the survey were likely to be located in the US, and thus results are only generalizable to other US medical librarians and not those of other nationalities or cultures [59].

Authorship is guided by international and professional standards, such as those from the ICMJE, and the American Psychological Association (APA). Some authorship criteria are stringent, and librarians surveyed for this study may not have met all the criteria for authorship when they enter authorship negotiations. For the respondents who reported they had been denied authorship, we did not assess the extent of librarian contributions to the research process or writing of the manuscript.

While this study examined the affective experiences of authorship among medical librarians, it cannot explain the reasons for the emotions experienced when negotiating authorship and when those emotions occurred in the negotiation process. The study also did not assess changes in affective experiences in authorship negotiations throughout a career trajectory, as we only collected data on what emotions were felt at any point in their career. It may be that affective experiences in authorship negotiations change as medical librarians gain experience and wisdom.

Also related to time factors, for our correlational analyses, we cannot say if the people who were first author on publications had published more because they had more support and appreciation from the research community or if they felt appreciated and supported by their research community because they had more publications.

The questionnaire also did not assess librarians' perceptions of other researchers' value of the library profession or how this may or may not affect negotiation approaches and outcomes. This is an important question that warrants further study.

## CONCLUSION

Librarians experience many emotions when asking for authorship including fear, sadness, dread, grief, happiness, joy, anxiety, frustration, and pride. For over 20 years librarianship has had an image problem, and librarians are undervalued by those around them, which may contribute to the fear, anxiety, dread, and other emotions experienced when asking for authorship [38]. More pride was felt when publishing with researchers outside of the LIS profession. If librarian research is already undervalued even by those within the profession, this can certainly translate over into librarians devaluing their own profession and thus not being able to negotiate as well with co-authors from other departments. Positively, participants in the survey reported feeling mostly supported and encouraged by their supervisors, research communities, and workplaces. The total number of articles or publications produced by those surveyed is correlated to certain aspects of research appreciation and support by their research communities. Lastly, librarians are still being denied authorship with other librarian colleagues and with colleagues on collaborative research projects taking place interdepartmentally, and librarians may need to push back harder for co-authorship credit when these situations arise.

## Data Availability

All datasets associated with this project are available at: https://osf.io/gdaet/?view_only=369a276fe415414d97145a6c431212ae.

## SUPPLEMENTAL FILES

**Appendix A:** Survey Questions

**Appendix B:** Group Statistics
