# Supplementary material for: Health sciences and medical librarians conducting research and their experiences asking for co-authorship
Source: J Med Libr Assoc. 2022 Oct 1;110(4):449–62. doi: 10.5195/jmla.2022.1485 (PMC10124612; doi:10.5195/jmla.2022.1485)
Supplement: Supplementary file 1 — Appendix A: Survey Questions [file jmla-110-4-449-s01.pdf]

Appendix A

Survey questions:

Do you serve health sciences or medical programs, health professionals and/or healthcare facilities in your capacity as a librarian?

- Yes
- No

Library experience

How many years have you worked in a library setting in any capacity (does not have to be as a professional librarian)? ___

How many years have you been working as a professional librarian or in an equivalent position? ___

- What is your designation at your current job?
- Administrative or professional staff
- Fixed term or non-tenured faculty
- Tenured faculty
- Tenure-track faculty, but not yet tenured
- Other, please describe:

What is your employment status currently?

- Full-time employment
- Part-time employment
- Other, please describe:

What is your type of library? Select the best choice:

- Academic Health Science Center / Academic Medical Center
- Academic (supporting health programs, but not at a health sciences or medical center)
- Association, Health or Medical- related
- Hospital / Health System (including government systems, e.g., Veterans Administration)
- Special (e.g. law, corporate, etc.)
- Other, please describe:

What is your role at your place of employment? Select all that apply.

- Liaison Librarian / Subject specialist / Academic librarian
- Solo Librarian
- Hospital Librarian
- Law or corporate librarian
- Collection development / Serials / Cataloger / Metadata specialist
- Access services / Circulation librarian
- Special collections / museum / archives librarian or archivist or specialist
- Systematic reviews librarian

- Other, please describe:

Training and field questions

Did you obtain a 4 year undergraduate degree?

- Yes
- No
- Other, please describe:

What is the broad disciplinary category of your undergraduate degree?

- Bachelor of Arts degree (B.A.)
- Bachelor of Science degree (B.S.)
- Bachelor of Fine Arts (B.F.A.)
- Bachelor of Social Work (B.S.W.)
- Bachelor of Engineering (B.Eng.)
- Bachelor of Science in Public Affairs (B.S.P.A)
- Bachelor of Science in Nursing (B.S.N.)
- Bachelor of Philosophy (B.Phil.)
- Bachelor of Architecture Degree (B.Arch.)
- Bachelor of Design (B.Des.)
- Other, please describe:

Did you obtain a library degree i.e. MLIS, MLS?

- Yes
- No

At what age did you obtain your library degree? ___

In your library degree program did you receive training on research methods?

- Yes
- No
- Unsure
- I don't have this degree.

Do you hold other advanced degrees outside of your library degree?

- Yes
- No

Select any other advanced degrees that you have outside of your library (master's level) degree.

- Second Masters degree in a subject area outside of library degree
- Doctorate in Library or Information Sciences
- Doctorate in medicine or health sciences - DDS, MD, DO
- Other doctorate degree

- Other health sciences degree
- Other, please describe:

In any other advanced Masters or Ph.D program besides the MLIS/MLS degree you hold, did you receive training on research methods?

- Yes
- No
- Unsure

Have you taken a statistics class at the undergraduate or graduate level?

- Yes
- No
- I don't hold a degree.

Have you already published research (this can include peer-reviewed journal articles, book chapters, books)?

- Yes
- No

What is the number of peer-reviewed articles, book chapters, or books you have published in your career as first author? ___

What is the number of peer-reviewed articles. book chapters, or books you have published in your career as an author in any other position in the author order other than first author? ___

What is the total number of peer-reviewed research articles, book chapters, or books you have had published? ___

Library support for research

Please answer the following questions by telling us how much you agree or disagree with the statements below about your current workplace:

I receive encouragement and personal attention from my supervisor(s).

I work in a library or setting that encourages research production.

- Strongly agree
- Agree
- Somewhat agree
- Neither agree nor disagree
- Somewhat disagree
- Disagree
- Strongly disagree

I feel that my supervisor(s) appreciate(s) my work.

- Strongly agree

- Agree
- Somewhat agree
- Neither agree nor disagree
- Somewhat disagree
- Disagree
- Strongly disagree

I can openly discuss any problems related to research with my supervisor(s).

- Strongly agree
- Agree
- Somewhat agree
- Neither agree nor disagree
- Somewhat disagree
- Disagree
- Strongly disagree

I feel accepted by my research community.

- Strongly agree
- Agree
- Somewhat agree
- Neither agree nor disagree
- Somewhat disagree
- Disagree
- Strongly disagree

I feel appreciated by my supervisor(s).

- Strongly agree
- Agree
- Somewhat agree
- Neither agree nor disagree
- Somewhat disagree
- Disagree
- Strongly disagree

I feel that the other members of my research community appreciate my work.

- Strongly agree
- Agree
- Somewhat agree
- Neither agree nor disagree
- Somewhat disagree
- Disagree
- Strongly disagree

There is a good sense of collegiality among the researchers I interact with.

- Strongly agree
- Agree
- Somewhat agree
- Neither agree nor disagree
- Somewhat disagree
- Disagree
- Strongly disagree

I receive encouragement and support from the other researchers.

- Strongly agree
- Agree
- Somewhat agree
- Neither agree nor disagree
- Somewhat disagree
- Disagree
- Strongly disagree

Collaboration on research with others

How do you think your colleagues or acquaintances describe your communication style on a daily basis? Please move the slider, with 1 being very passive, 7 being very assertive (1-7 Likert scale).

- Passive Communication is a style in which individuals are more cautious about expressing thoughts and feelings openly.
- Assertive Communication is a style in which individuals openly express their thoughts and feelings.

The following questions deal with authorship opportunities with other librarians, library staff, or library workers.

As a librarian, have you collaborated on research with other librarians, library staff, or library workers and asked for authorship?

- Yes
- No

Please rate the extent to which you have experienced the following emotions when asking for authorship with other librarians, library staff, or library workers. 1 indicates experienced the emotion in a small degree, 3-4 means have experienced a moderate amount of this emotion at any point in time, 7 means experienced the emotion to the fullest extent, at any point in time of asking for authorship. Please select N/A if you never experienced it. (1-7 Likert scale)

- Fear
- Anxiety
- Sadness

- Worry
- Dread
- Happiness
- Excitement
- Pride
- Frustration
- Anger
- Disgust
- Devastation
- Grief or loss
- Joy
- Eagerness
- Optimism
- Stress
- Anticipation
- Hope
- Guilt

As a librarian, were you ever given or offered authorship on research projects with other librarians, library staff, or library workers without having to ask to be included as an author? Please only answer "yes" if you've been offered authorship for merited situations where you made a substantial contribution to the publication.

- Yes
- No

Have you ever been refused authorship on a publication with other librarians, library staff, or library workers?

- Yes
- No

If so, how many times have you been refused authorship on a publication with other librarians, library staff, or library workers? ___

The following questions deal with authorship opportunities with faculty members orc olleagues you might publish with. These questions do not include research with other librarians, library staff, or library workers.

As a librarian, have you collaborated on research with anyone who is not another librarian, library staff, or library workers and asked for authorship?

- Yes
- No

Please rate the extent to which you have experienced the following emotions when asking for authorship with anyone who is not another librarian, library staff, or library workers. 1 indicates

experienced the emotion in a small degree, 3-4 means have experienced a moderate amount of this emotion at any point in time, 7 means experienced the emotion to the fullest extent, at any point in time of asking for authorship. Please select N/A if you never experienced it. (1-7 Likert scale)

- Fear
- Anxiety
- Sadness
- Worry
- Dread
- Happiness
- Excitement
- Pride
- Frustration
- Anger
- Disgust
- Devastation
- Grief or loss
- Joy
- Eagerness
- Optimism
- Stress
- Anticipation
- Hope
- Guilt

As a librarian, were you ever given or offered authorship with anyone who is not another librarian, library staff, or library workers without having to ask to be included as an author?

- Yes
- No

Have you ever been refused authorship on a publication with anyone who is not another librarian, library staff, or library workers?

- Yes
- No

If so, how many times have you been refused authorship on a publication with anyone who is not another librarian, library staff, or library workers? ___

Demographic Questions

What is your age? ___

Are you of Hispanic, Latino, or Spanish origin?

- Yes
- No

- Other, please specify:
- Prefer not to answer

How would you best describe yourself? You can select multiple options.

- American Indian or Alaska Native. A person having origins in any of the original peoples of North
- and South America (including Central America), and who maintains tribal affiliation or community
- attachment.
- Asian. A person having origins in any of the original peoples of the Far East, Southeast Asia, or
- the Indian subcontinent including, for example, Cambodia, China, India, Japan, Korea, Malaysia,
- Pakistan, the Philippine Islands, Thailand, and Vietnam.
- Black or African American. A person having origins in any of the black racial groups of Africa.
- Terms such as "Haitian" can be used in addition to "Black or African American."
- Native Hawaiian or Other Pacific Islander. A person having origins in any of the original peoples
- of Hawaii, Guam, Samoa, or other Pacific Islands.
- White. A person having origins in any of the original peoples of Europe, the Middle East, or
- North Africa.
- Prefer to self-describe:
- Prefer not to answer

What is your current gender identity?

- Male
- Female
- Female-to-male (FTM)/Transgender Male/Trans Man
- Male-to-Female (MTF)/Transgender Female/Trans Woman
- Genderqueer, neither exclusively male nor female
- Additional Gender Category/(or Other), please specify:
- Prefer not to answer

What sex were you assigned at birth on your original birth certificate (Check one)

- Male
- Female
- Prefer not to answer
