# Supplementary material for: Health sciences and medical librarians conducting research and their experiences asking for co-authorship
Source: J Med Libr Assoc. 2022 Oct 1;110(4):449–62. doi: 10.5195/jmla.2022.1485 (PMC10124612; doi:10.5195/jmla.2022.1485)
Supplement: Supplementary file 2 — Appendix B: Group Statistics [file jmla-110-4-449-s02.pdf]

Appendix B

Group Statistics – The data in this table corresponds to "Librarian Emotions When Negotiating Authorship" (Figure 1 in text)

	Group	N	Mean	Std. Deviation	Std. Error Mean
FEAR	With Library Colleagues	197	61.57	47.358	3.374
	With Non-Librarians	214	27.94	41.913	2.865
ANXIETY	With Library Colleagues	197	46.74	48.061	3.424
	With Non-Librarians	215	76.03	41.289	2.816
SADNESS	With Library Colleagues	197	76.86	40.803	2.907
	With Non-Librarians	216	44.35	47.643	3.242
WORRY	With Library Colleagues	197	52.95	48.331	3.443
	With Non-Librarians	215	58.21	47.734	3.255
DREAD	With Library Colleagues	196	61.49	47.262	3.376
	With Non-Librarians	215	32.10	43.193	2.946
FRUSTRATION	With Library Colleagues	196	46.73	47.807	3.415
	With Non-Librarians	215	71.15	43.864	2.991
ANGER	With Library Colleagues	195	75.11	41.925	3.002

	With Non-Librarians	214	78.14	39.956	2.731
DISGUST	With Library Colleagues	195	79.10	39.275	2.813
	With Non-Librarians	215	81.35	37.590	2.564
DEVASTATION	With Library Colleagues	194	81.43	37.542	2.695
	With Non-Librarians	215	81.31	37.677	2.570
GRIEF	With Library Colleagues	195	81.03	37.875	2.712
	With Non-Librarians	216	41.46	46.477	3.162
STRESS	With Library Colleagues	196	34.67	44.931	3.209
	With Non-Librarians	215	29.20	42.050	2.868
GUILT	With Library Colleagues	195	70.22	44.360	3.177
	With Non-Librarians	213	72.19	43.427	2.976
HAPPINESS	With Library Colleagues	266	29.38	41.704	2.557
	With Non-Librarians	215	21.14	36.192	2.468
EXCITEMENT	With Library Colleagues	197	25.78	39.393	2.807
	With Non-Librarians	216	20.86	35.642	2.425
PRIDE	With Library Colleagues	196	24.07	38.069	2.719
	With Non-Librarians	214	40.34	46.822	3.201

JOY	With Library Colleagues	196	40.06	46.041	3.289
	With Non-Librarians	216	23.15	37.934	2.581
EAGERNESS	With Library Colleagues	196	27.26	40.423	2.887
	With Non-Librarians	216	21.03	36.145	2.459
OPTIMISM	With Library Colleagues	196	21.95	36.674	2.620
	With Non-Librarians	216	29.35	42.324	2.880
ANTICIPATION	With Library Colleagues	196	31.05	43.109	3.079
	With Non-Librarians	214	35.69	44.737	3.058
HOPE	With Library Colleagues	196	31.19	43.022	3.073
	With Non-Librarians	213	72.19	43.427	2.976
